# A genetical metabolomics approach for bioprospecting plant biosynthetic gene clusters

**DOI:** 10.1186/s13104-019-4222-3

**Published:** 2019-04-02

**Authors:** Lotte Witjes, Rik Kooke, Justin J. J. van der Hooft, Ric C. H. de Vos, Joost J. B. Keurentjes, Marnix H. Medema, Harm Nijveen

**Affiliations:** 1 Bioinformatics Group, Wageningen University & Research, Wageningen, The Netherlands; 2 Laboratory of Genetics, Wageningen University & Research, Wageningen, The Netherlands; 3 Business Unit Bioscience, Wageningen University & Research, Wageningen, The Netherlands; 4 Centre for BioSystems Genomics, Wageningen, The Netherlands; 5 Netherlands Metabolomics Centre, Utrecht, The Netherlands

**Keywords:** Bioinformatics, Specialized metabolism, Natural products, Gene cluster, Genetics, GWAS, QTL, Metabolomics, Mass spectrometry, Comparative genomics

## Abstract

**Objective:**

Plants produce a plethora of specialized metabolites to defend themselves against pathogens and insects, to attract pollinators and to communicate with other organisms. Many of these are also applied in the clinic and in agriculture. Genes encoding the enzymes that drive the biosynthesis of these metabolites are sometimes physically grouped on the chromosome, in regions called biosynthetic gene clusters (BGCs). Several algorithms have been developed to identify plant BGCs, but a large percentage of predicted gene clusters upon further inspection do not show coexpression or do not encode a single functional biosynthetic pathway. Hence, further prioritization is needed.

**Results:**

Here, we introduce a strategy to systematically evaluate potential functions of predicted BGCs by superimposing their locations on metabolite quantitative trait loci (mQTLs). We show the feasibility of such an approach by integrating automated BGC prediction with mQTL datasets originating from a recombinant inbred line (RIL) population of *Oryza sativa* and a genome-wide association study (GWAS) of *Arabidopsis thaliana*. In these data, we identified several links for which the enzyme content of the BGCs matches well with the chemical features observed in the metabolite structure, suggesting that this method can effectively guide bioprospecting of plant BGCs.

**Electronic supplementary material:**

The online version of this article (10.1186/s13104-019-4222-3) contains supplementary material, which is available to authorized users.

## Introduction

Plant specialized metabolism is the source of hundreds of thousands of natural products. These molecules play key roles in plant development and ecology as, e.g., defense agents and signals, and are broadly applied as medicines, dyes, flavorings and cosmetics. With the sequencing of hundreds of plant genomes, genome mining has become a new strategy to uncover the biosynthetic pathways towards known molecules of interest as well as to identify pathways towards novel compounds [1]. The recent discovery that significant numbers of plant metabolic pathways are encoded by physically clustered genes further facilitates the genome mining process, as it enables rapid identification of candidate pathways from genome sequences alone [2]. Multiple tools have become available that automate the identification of these biosynthetic gene clusters (BGCs) in plant genomes [3–5]. Moreover, synthetic biology platforms have been developed for (transient) heterologous expression of such gene clusters in, e.g., tobacco and yeast, which allows relatively fast experimental exploration of plant’s biosynthetic potential [6–8].

However, heterologous expression of BGCs still entails a significant amount of work. Moreover, it appears that a substantial proportion of predicted gene clusters may not be *bona fide* BGCs; in such cases, multiple enzyme-coding genes—while located adjacently on the chromosome and therefore triggering BGC prediction—do not in fact encode subsequent catalytic steps in one and the same pathway, and their transcription is not co-regulated. Indeed, Wisecaver et al. reported limited overlap between BGCs and coexpression modules they obtained from large-scale transcriptomics data [9] (although they predicted these BGCs with methods not specifically designed for plants). Similarly, Kautsar et al. found that strong coexpression within a BGC could be detected for around 25% of the gene clusters predicted in *Arabidopsis thaliana* [4]. They identified two cases in which enzyme-coding genes within predicted BGCs clearly encoded unrelated steps in glucosinolate biosynthesis.

Hence, to capitalize on plant BGCs for the discovery of natural products and their pathways, new methods are required to prioritize predicted gene clusters. Besides transcriptomic analysis, another promising avenue for this is the combined use of metabolomics and genetic data to systematically connect gene clusters with known and yet unknown metabolites based on natural variation [10, 11]. Indeed, several recent genetic studies in different plant species use untargeted metabolomics of plant populations to associate metabolite abundance quantitative trait loci (mQTLs) to enzyme-coding genes [12–16].

Here we argue that such metabolomics-based systems genetics approaches can be extended to systematically study plant BGCs and prioritize them for heterologous expression. To illustrate this, we use datasets from a recombinant inbred line (RIL) population from *Oryza sativa* and a genome-wide association study (GWAS) from *Arabidopsis* to establish a proof of principle, showing that studying the overlap of mQTLs from such data with predicted BGCs generates interesting hypotheses regarding the functional significance of these putative gene clusters.

## Main text

**Fig. 1:**
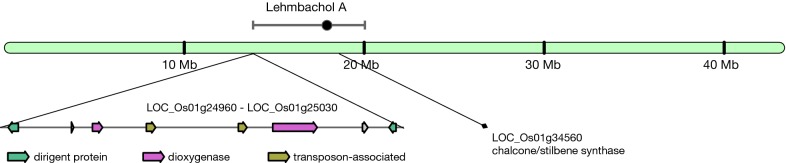
Overlap between a predicted lignan BGC and an mQTL for lehmbachol A on rice chromosome 1. An unclustered chalcone/stilbene synthase-encoding gene (LOC_Os01g34560) is located further downstream within the same mQTL region

To assess the potential of identifying mQTL-BGC overlaps, we first used an mQTL dataset from an *O. sativa* RIL population (germinating seeds and leaves) published by Gong et al. [12]. These mQTLs were overlaid with BGCs predicted by plantiSMASH [4] (see Additional file 1). In this dataset, we identified a possible link between a predicted lignan BGC on chromosome 1 (14,059,096–14,124,875 bp) and an mQTL for lehmbachol A (LOD-score: 3.3; Fig. 1). We could not trace any literature reporting on the biosynthesis of this molecule. Interestingly, it is a hydroxylated stilbenolignan compound, which matches well with the combination of genes found in the BGC: two dirigent enzymes (known to be responsible for directing the key C–C coupling step in lignan biosynthesis) and two dioxygenases that could hydroxylate the lignan scaffold. One of the dioxygenases (LOC_Os01g25010, expressed in leaves) had three non-synonymous SNPs in the dioxygenase domain, which might be causal for the variation in the lehmbachol A production leading to the mQTL. The other dioxygenase (LOC_Os01g24980) shows strong coexpression with one of the dirigent enzymes (LOC_Os01g25030) in root samples (see Additional file 2: Figure S1). Further downstream within the same mQTL region (and close to its peak, see Fig. 1), a chalcone/stilbene synthase-encoding gene is also present (LOC_Os01g34560), which is expressed in shoots and leaves according to the Rice Expression Database [17]. There is a second mQTL for lehmbachol A as well, located on chromosome 10. In this region, another chalcone/stilbene synthase-coding gene (LOC_Os10g28060) can be found, which is clustered with nearby genes for a hydrolase (LOC_Os10g28020), an acetyltransferase (LOC_Os10g28040) and, somewhat further downstream, an epimerase (LOC_Os10g28200). It is not unthinkable that this second locus is (also) involved in the biosynthesis of lehmbachol. Both these two putative biosynthetic loci would represent interesting candidates for further study.

Another interesting overlap in this rice dataset was found between a predicted polyketide BGC on chromosome 11 (18,762,365–18,822,272 bp) and several flavonoid mQTLs, including an mQTL for putatively identified isogemichalcone B (LOD-score: 4.5). The flavonoid mQTLs match well with the presence of chalcone/stilbene synthases in the predicted BGC.

We also used an mQTL dataset from a GWAS study with 349 *A. thaliana* accessions genotyped at 214,051 markers (see Additional file 1). Unbiased metabolomics was performed with accurate mass LC–MS on full rosette leaf tissue grown under normal conditions, and raw MS spectral data were processed with MetAlign-MSClust [18]. Linear mixed models in EMMA [19] and GAPIT [20] were applied to the genotype and metabolite profiling matrix, resulting in 1897 significant mQTLs (see Additional file 1). Again, the mQTLs were overlaid with BGCs predicted by plantiSMASH [4].

By examining the mQTL-BGC overlaps in the *Arabidopsis* dataset (see Additional file 1), we identified several cases in which predicted BGCs overlapped with mQTLs corresponding to molecules which are in fact known to be synthesized by enzymes in specialized metabolic pathways encoded by non-clustered genes. For example, a putative saccharide BGC located on chromosome 2 (9,744,720–9,841,503 bp) overlapped with multiple mQTLs connected to flavonoid saccharides, including kaempferitrin, a kaempferol species that is *O*-rhamnosylated on the third and seventh carbon atoms. The predicted BGC contained a UDP-glycosyltransferase (AT2G22930), which is similar in sequence to quercetin 3-*O*-glucosyltransferases. It is possible that this glycosyltransferase is able to 3-*O*-rhamnosylate kaempferol, since kaempferol and quercetin only differ in one hydroxy group on the B-ring. Alternatively, if the UDP-glycosyltransferase only has substrate specificity for glucose and not rhamnose, the mQTL could be caused by an indirect effect, due to glucosylation competing with rhamnosylation of the same flavonoid substrate. Intriguingly, the predicted gene cluster also encodes multiple Scl acyltransferases, two of which (AT2G22990 and AT2G23000) have previously been shown to act as anthocyanin sinapoyltransferases [21]. We observed a strong degree of coexpression (Pearson correlation coefficients of > 0.79) for the glycosyltransferase AT2G22930 with three Scl acyltransferases (AT2G22920, AT2G22960 and AT2G23000) in a leaf time-course analysis of the response to barley powdery mildew fungus Bgh (NCBI GEO dataset GSE39463, Additional file 2: Figure S2). Altogether, this result suggests that multiple enzymes encoded in this predicted gene cluster are involved in different types of flavonoid modification. We also found mQTLs for kaempferitrin in three other loci, encoding Scl acyltransferases, a cytochrome P450 and a beta-glucosidase that may potentially be involved in further modifying or breaking down the molecule. While this locus thus does not seem to encode a complete biosynthetic pathway by itself, it is still likely to encode multiple enzymatic steps involved in the same pathway, and may represent a case of ‘partial’ pathway clustering similar to cases reported for monoindole terpene alkaloid biosynthesis in *Catharanthus roseus* [22].

Of the four experimentally characterized BGCs in *Arabidopsis*, three—the thalianol, marneral and tirucalladienol clusters—are specifically expressed in roots [23–25]; hence, we did not expect to find mQTLs for these molecules in this GWAS dataset. The fourth, the arabidiol/baruol BGC, contains some genes that are expressed in both root and leaf (such as the baruol synthase PEN2, according to the *Arabidopsis* eFP browser [http://bar.utoronto.ca/efp/cgi-bin/efpWeb.cgi]). Indeed, six mQTLs mapped to different genes in this BGC, and two of these mQTLs mapped specifically to the PEN1 and PEN2 oxidosqualene cyclase-encoding genes. The masses of the metabolites connected to this mQTL represent yet unknown compounds, and further research (e.g. MS/MS fragmentation analysis) is needed to confirm whether these masses belong to arabidiol/baruol derivatives or unrelated metabolites due to, e.g., downstream effects.

We also observed cases in which predicted BGCs may be regarded as putative false positives, in the sense that they do not encode enzyme-coding genes that are likely to function within the same pathway. E.g., a methoxyglucobrassicin mQTL was associated with the cytochrome P450 gene CYP81F2 (AT5G57220, which has been implicated in glucobrassicin biosynthesis [26]) within a predicted BGC (chromosome 5: 23,184,526–23,213,996 bp) containing no other enzyme-coding genes known to be involved in glucosinolate biosynthesis. For two other methoxyglucobrassicin mQTLs we could not derive a functional link with glucosinolate biosynthesis.

Finally and perhaps most interestingly, 23 predicted gene clusters showed only overlap with mQTLs of metabolites that have not been annotated yet, showing a clear potential for discovery of novel enzymes that can be tested through synthetic biology approaches to identify novel chemistry. Among such predicted gene clusters, one may identify likely *bona fide* BGCs by finding cases in which multiple mQTLs overlap with the same predicted BGC (and in the case of GWAS, with multiple different genes within it) and are likely to represent biosynthetically connected molecules, based on e.g. having defined mass differences between them.

Of course, it is also possible to look for overlap of mQTLs with enzyme-coding genes in a non-BGC-centric fashion. By means of example, we scanned all *Arabidopsis* mQTLs for enzyme families known to be involved in biosynthetic pathways using all profile Hidden Markov Models from plantiSMASH [4] that are related to scaffold biosynthesis. While the results (see Additional file 1) include some potentially interesting links (e.g., linking TPS04 with a cyclohexene-related terpene and linking TPS08 with a naphthalene-related terpene), these mQTLs may also be caused by indirect effects, e.g. through affecting precursor pools, especially given the fact that these terpene synthases have been linked to the production of different terpenes in other studies [27, 28].

Our results confirm that indeed (genes in) predicted BGCs can be meaningfully linked to mQTLs explaining variation in their metabolic products by GWAS and RIL-based studies, allowing the prioritization of BGCs for further analysis and the generation of new hypotheses about the functions of these predicted BGCs. Altogether, we conclude that the prediction of BGCs in plant genomes for bioprospecting can become a more powerful tool for discovery when complemented not only with coexpression analysis but also with unsupervised metabolomics linked to genetic variation. Most importantly, this allows identifying predicted BGCs that overlap with genomic loci associated with the abundance of unknown molecules, which would constitute candidates for further investigation. At the same time, this makes it possible to distinguish these from genomic loci involved in the biosynthesis of well-known molecules whose biosynthetic pathways are known not to be clustered.

Thus, the metabolomics-based systems genetics approach described here has the potential to become an important technology for the systematic genome-wide assessment of biosynthetic genes that can be prioritized for heterologous expression using the latest synthetic biology methodologies [7].

## Limitations

Although we were able to predict several links between BGCs and metabolites, we did not have the resources to experimentally validate these links through mutagenesis or heterologous expression. It is possible that some mQTLs in fact represent indirect effects. Also, the RIL data from rice resulted in relatively broad mQTL regions, in which other genes may be hidden that could be causative of the metabolic differences underlying the QTLs.

Additionally, the metabolomics datasets were limited to shoots and leaves, while many key metabolites may be specifically produced in roots. Using a larger number of relevant conditions in the future (including root metabolomics and samples from biotic or abiotic stress treatments) will make it possible to connect metabolites to gene clusters and gene cluster-like genomic loci on larger scales.

Finally, in the *Arabidopsis* metabolomics study applied here, we did not generate dedicated metabolite fragmentation data, such as MS/MS and MS^n^ spectra [29], making it yet difficult to predict the nature of the unknown metabolites linked to putative BGCs of unknown function. New technologies that generate and exploit metabolite fragmentation data based on molecular networking and substructure identification [30–32] will make it easier to obtain structural information for such unknowns, and thus facilitate assessing whether the combination of molecular and genetic (enzymatic) features observed in an mQTL-BGC pair shows high potential or not.

## Additional files

**Additional file 1.** List of predicted BGCs, list of mQTLs, list of BGCs with mQTLs, list of matches for mQTL genes with profile Hidden Markov Models related to scaffold biosynthesis, overview of LCMS clusters.

**Additional file 2: Figure S1.** Coexpression analysis of two dioxygenase-coding genes and two dirigent-enzyme-coding genes on rice chromosome 1. **Figure S2.** Coexpression analysis of the kaempferitrin-associated predicted BGC in *Arabidopsis thaliana*.

## Abbreviations

BGC: biosynthetic gene cluster
mQTL: metabolite quantitative trait locus
RIL: recombinant inbred line
GWAS: genome-wide association study
LOD: logarithm of the odds ratio
SNP: single nucleotide polymorphism
LC: liquid chromatography
MS: mass spectrometry
