# Supplementary material for: A genetical metabolomics approach for bioprospecting plant biosynthetic gene clusters
Source: BMC Res Notes. 2019 Apr 2;12:194. doi: 10.1186/s13104-019-4222-3 (PMC6444639; doi:10.1186/s13104-019-4222-3)
Supplement: Supplementary file 2 — Additional file 2: Figure S1. Coexpression analysis of two dioxygenase-coding genes and two dirigent-enzyme-coding genes on rice chromosome 1. Figure S2. Coexpression analysis of the kaempferitrin-associated predicted BGC in Arabidopsis thaliana. [file 13104_2019_4222_MOESM2_ESM.docx]

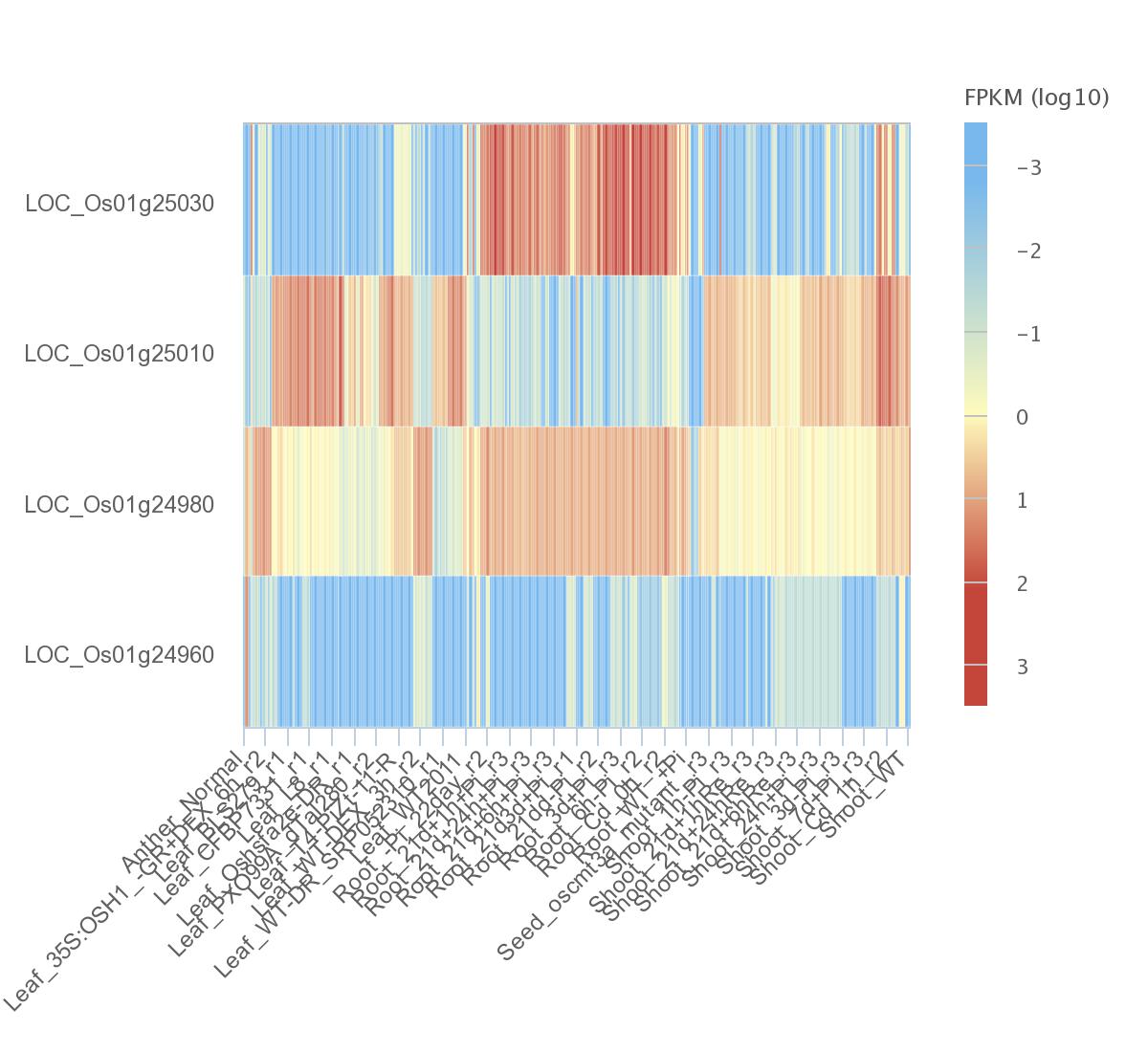


**Figure S1.** Coexpression analysis from the Rice Expression Database (<http://expression.ic4r.org/>) of two dioxygenase-coding genes and two Dirigent-enzyme-coding genes on rice chromosome 1. One Dirigent-enzyme -coding gene (LOC_Os01g25030) appears coexpressed with one dioxygenase-coding gene (LOC_Os01g24980), while the second dioxygenase-coding gene (LOC_Os01g25010) shows an anti-correlated expression pattern. The second Dirigent-enzyme-coding gene (LOC_Os01g24960) is not expressed in the data.


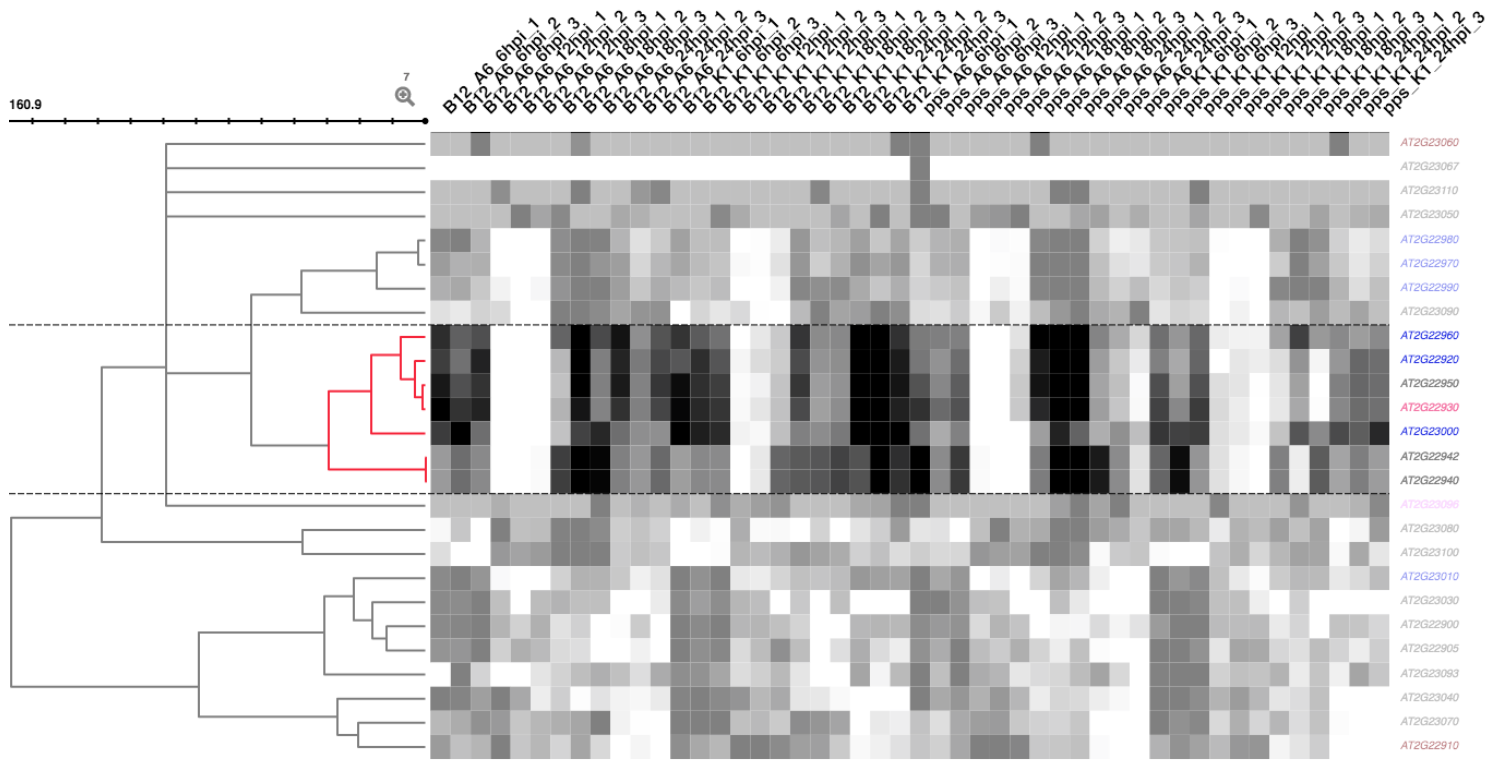


**Figure S2.** PlantiSMASH coexpression analysis of the kaempferitrin-associated predicted BGC in *Arabidopsis* thaliana, using NCBI GEO dataset GSE39463, a time-course leaf RNA-seq analysis of the response to barley powdery mildew fungus Bgh. The glycosyltransferase gene (AT2G22930) shows a strong degree of coexpression (Pearson correlation coefficient of >0.79) with several Scl acyltransferase-coding genes (AT2G22920, AT2G22960 and AT2G23000).
